# Improved Interfacial Contact for Pyramidal Texturing of Silicon Heterojunction Solar Cells

**DOI:** 10.3390/molecules27051710

**Published:** 2022-03-05

**Authors:** Ruijie Dai, Tengzuo Huang, Weijie Zhou, Jinpeng Yang, Hua Zhang, Fayin Yu, Anran Chen, Feng Wang, Jin Zhang, Tao Sun, Longzhou Zhang

**Affiliations:** 1 International Joint Research Center for Optoelectronic and Energy Materials, Yunnan University, Kunming 650091, China; ruijie.dai@mail.ynu.edu.cn (R.D.); htzireael@mail.ynu.edu.cn (T.H.); weijiezhou@mail.ynu.edu.cn (W.Z.); yangjinpeng@mail.ynu.edu.cn (J.Y.); zhanghua1226@mail.ynu.edu.cn (H.Z.); 15008613536@163.com (F.Y.); wangfeng123@mail.ynu.edu.cn (F.W.); 2 School of Materials and Energy, Yunnan University, Kunming 650091, China

**Keywords:** thin films, optical materials and properties, interfaces, heterojunction solar cells

## Abstract

Reducing the surface reflectivity of silicon substrates is essential for preparing high-performance Si-based solar cells. We synthesized pyramid-nanowire-structured Si (Si-PNWs) anti-reflection substrates, which have excellent light-trapping ability (<4% reflectance). Furthermore, diethyl phthalate (DEP), a water-insoluble phthalic acid ester, was applied to optimize the Si-PNWs/PEDOT:PSS interface; the photoelectric conversion efficiency of heterojunction solar cells was shown to increase from 9.82% to 13.48%. We performed a detailed examination of the shape and optical characteristics of Si-PNWs, as well as associated photoelectric performance tests, to investigate the origin of performance improvements in Si-PNWs/PEDOT:PSS heterojunction solar cells (HSCs).

## 1. Introduction

Si/poly(3,4-ethylenedioxythiophene)-poly(styrenesulfonate) (PEDOT:PSS) heterojunction solar cells (HSCs) are promising candidates for commercial photovoltaic cells, as they can be fabricated using simple low-cost preparation techniques and achieve high output efficiency [1,2,3,4,5]. Previous studies have shown that the typical average reflectivity of traditional pyramid-textured Si substrates exceeds 12% in the 400–1000 nm wavelength range [6,7,8,9,10]. Owing to the visible light reflection characteristics of silicon nanowires (Si-NWs), combinatorial pyramid-nanowire Si substrates (denoted as Si-PNWs) offer several advantages in solar cells applications. The contact area between PEDOT:PSS and the Si substrate interface is increased, the high surface topography of villiform can significantly enhance the absorption of sunlight [11,12,13,14,15,16,17]. It was demonstrated that the pyramid form influenced incident light reflection and heterojunction interface properties [11,12,18,19]. However, detailed investigations of unique three-dimensional micro-nano-textured substrates applied in Si/organic HSCs are rare. It is still unclear if the texture with tiny pyramids combined with nanowires can increase the efficiency of Si/organic HSCs. It is critical to explore the effect of texturing pyramids with different-sized nanowires on the performance of the Si/organic HSCs.

In this work, we applied the chemical etching method to prepare Si-PNWs. After optimizing the synthesis procedure, the experimental results showed that our novel combinatorial Si substrates exhibited improved light absorption relative to Si substrates with a traditional micro-nano structure. In addition, the proposed substrates enhanced the photoelectric performance of Si/organic heterojunction solar cells, which might pave the way for Si/PEDOT:PSS HSCs to be manufactured on a large scale.

## 2. Materials and Methods

The n-type Si wafers (1–3 Ω·cm, <100>, 275 ± 10 μm) were cleaned using the RCA standard method. After removing the native oxide on the surfaces by a 10 wt% hydrofluoric acid (HF) solution, the polished side of the wafer was textured into pyramids structure by immersing in a mixture of 4.5 wt% KOH and 18 vol% IPA solutions at 85 °C for 30 min. Next, immersing in different concentrations of AgNO_3_ (1–9 mM) and 4.5 mM HF mixed solution for 2 min to deposit a layer of silver particles. Next, etching the samples in a mixed water solution of 4.5 mM HF, 40 mM H_2_O_2_ at room temperature for 3 min, the residual Ag nanoparticles on the nanotextured surfaces were removed by dipping in nitric acid and cleaning and blow-drying with nitrogen. The 100 nm Al was thermally evaporated as a negative electrode and transferred to a glove box. PEDOT:PSS (Clevios PH-1000) solution was added with 5 wt% dimethyl sulfoxide (DMSO) and 0.2 wt% Triton X-100 and stirred overnight. This mixed solution was dropped evenly onto the Si-PNWs pyramids for 20 s, then spin-coated at 2000 rpm for 40 s, followed by annealing at 120 °C for 30 min. Then, 200 nm Ag-grids were thermally evaporated as an anode. Diethyl phthalate (DEP) was spin-coated upon the Ag-grids at 5000 rpm for 60 s, then annealed at 120 °C for 10 min. All the characterizations involved are displayed in the Supplementary Materials.

## 3. Results and Discussion

**Figure 1:**
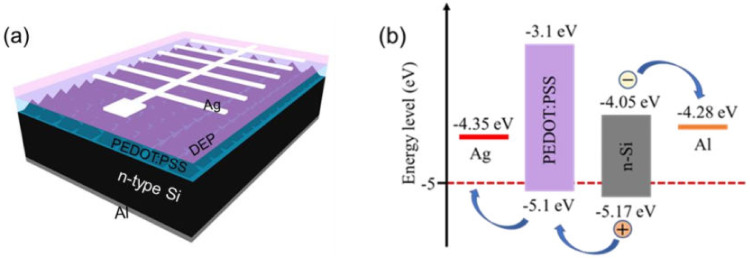
(**a**) Structure diagram of the Si-PNWs/PEDOT:PSS solar cells with DEP coating. (**b**) Energy band diagrams of the Si-PNWs/PEDOT:PSS solar cells.

**Figure 2:**
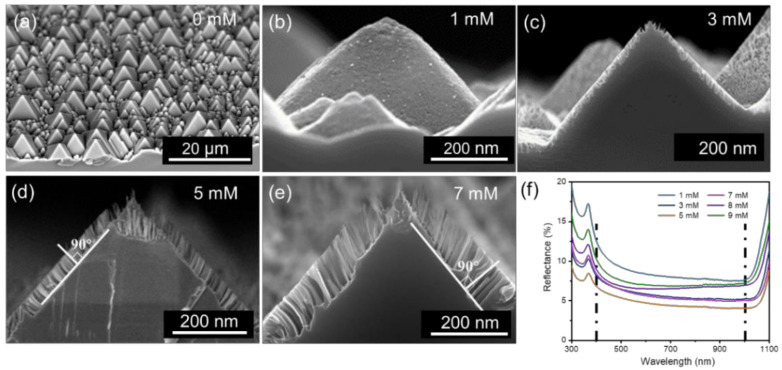
The devices produced using different AgNO_3_ concentrations (**a**–**e**) SEM images. (**f**) Reflectance.

We successfully fabricated novel Si-PNWs textured substrates. The device’s structure diagram and energy band diagrams are shown in Figure 1a,b, respectively. The morphology of Si-PNWs was analyzed by scanning electron microscopy (SEM). As shown in Figure 2a, pure pyramidal texturing exhibited a relatively smooth surface. In Figure 2b, 1 mM AgNO_3_ transformed the original surface from smooth to rough. Figure 2c reveals the Si-PNWs composite structure that emerges when AgNO_3_ is increased to 3 mM. At an AgNO_3_ concentration of 5 mM, the nanowires grew perpendicular to the pyramid structure, with a length of approximately 50 nm (Figure 2d). As the AgNO_3_ increased to 7 mM, the nanowires continued to grow longer, which could weaken the light trapping abilities that are key to enhancing the performance of the solar cells (Figure 2e). When the concentration was increased to 9 mM, the Si-PNWs become irregular and partially destroyed. The SEM images indicate that the nanowires prepared using 5 mM AgNO_3_ exhibited intact structures, with nanowires of suitable lengths. The reflectance was measured to characterize the antireflection characteristics of the Si-PNWs. The sample prepared using 5 mM AgNO_3_ aqueous solution showed an average reflectance of 4.68%. The reflectance spectra of different texture structures of silicon are shown in Figure S3 and Figure 2e. The gradual decrease in reflectance corresponds to an increase in photon absorption, thereby increasing J_SC_. As the nanowires grow, the FF decreases, causing the PCE to start declining. AgNO_3_ (5 mM) yields a PCE as high as 12.18%; however, the FF is only 60.98%. We believe that this is due to the surface tension of the Si/PEDOT:PSS interface leading to poor interface contact. To address this issue, we chose the HSCs made of 5 mM AgNO_3_ as a reference device, then the diethyl phthalate (DEP) was introduced on this device for modification. The DEP coating is expected to promote interface optimization [18,20].

**Figure 3:**
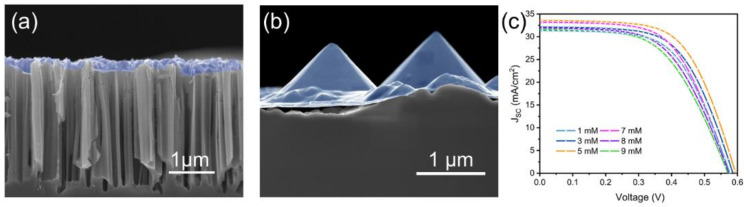
(**a**,**b**) SEM images of silicon nanowires and pyramids (**c**) J–V curves for the AgNO_3_ treated Si-PNWs solar cells.

It was difficult for organic matter to penetrate from the SiNWs’ surface to the bottom due to the high density and the coverage was also uneven by comparing the contact between two texture structures and PEDOT: PSS (see the SEM in Figure 3a). The PEDOT layer near the tips of the SiNWs in this structure is very thin. This will lead to a low shunt resistance and thus reduced Voc and FF. The drop in Voc for cells with longer SiNWs is also attributed to higher carrier recombination associated with their increased surface area [21,22,23,24]. The defective surface of the SiNWs prepared by electroless etching and the poor infiltration and coverage of PEDOT onto the SiNW surface will exacerbate carrier recombination and lower the Voc. As compared to the SiNW cell, the SEM in Figure 3b reveals that the PEDOT solution tends to flow down to the bottom edges of the base of the pyramid due to gravitational force. The organic material is in close contact with the porous silicon pyramid structure, and the thickness of the PEDOT film is homogeneous, which is important for improving hybrid solar cell device stability and conversion efficiency. Figure 3c shows the J–V characteristics curves of the HSCs fabricated using different AgNO_3_ concentrations, in which related performance parameters (short circuit current (J_SC_), open-circuit voltage (V_OC_), fill factor (FF), photoelectric conversion efficiency (PCE)), and from 300–1000 nm are summarized in Table 1. The light absorption was enhanced after nanowire-structure was constructed. The photoelectric properties and the average reflectance values of the differently textured silicon solar cells are listed in Table 1.

**Figure 4:**
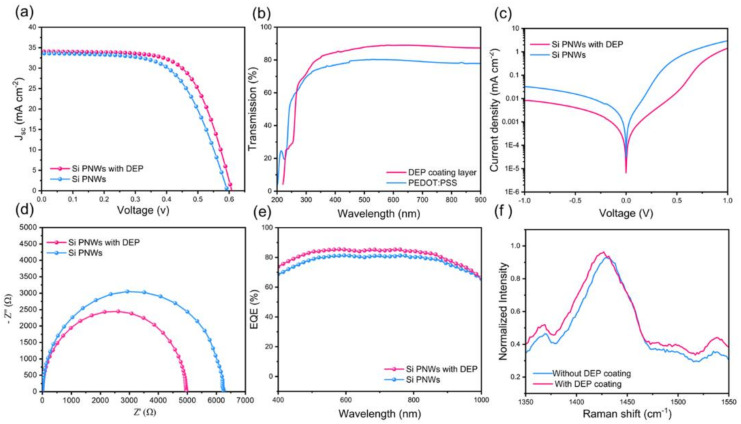
The devices coated with and without DEP: (**a**) J–V curves. (**b**) Reflectance. (**c**) Dark J-V curves. (**d**) Electrochemical impedance spectroscopy (EIS). (**e**) EQE. (**f**) Raman spectrum.

Experimental results prove that the best device with DEP coating achieves a PCE of 13.48% and an FF of 65.11%. The current-voltage curve in Figure 4a shows that the addition of the DEP coating increases the PCE of the device from 12.08% to 13.48%; improving the performance of the device is mainly due to the increase in FF from 60.98% to 65.11% (Table 2). We prepared thin films of DEP and PEDOT:PSS on quartz glass to study their optical transmittance. As shown in Figure 4b, the transmittance of DEP coating is significantly better than that of PEDOT:PSS, implying that the introduction of DEP coating would not reduce the light absorption of the HSCs. As shown in Figure 4c, the introduction of DEP coating significantly reduces the reverse saturation current (J_o_), the J_o_ can be considered to be closely related to the recombination of photogenerated carriers, and a smaller J_o_ induces a higher J_SC_ and V_oc_. The reduction in J_o_ means an improvement in the quality of the surface contact between the PEDOT:PSS thin films and the textured structure, and the defect density of the junction interface is reduced. The addition of coating layers caused conformal contact between the PEDOT: PSS film and the pyramid-textured PNWs surface, reducing interfacial re-combination losses and improving the carrier collecting capability, enhancing the charge dissociation and extraction abilities, and resulting in synergies in Voc enhancement [25,26,27]. In order to further prove the influence of the introduction of the DEP coating on the Si/PEDOT:PSS interface, electrochemical impedance was measured, as shown in Figure 4d. The substantial increase in electrochemical impedance proves that the quality of the heterojunction interface has been enhanced, which is consistent with the increase in the FF and the decrease in the reverse saturation current density [28,29,30,31]. As shown in Figure 4e, the improvement in quantum efficiency (EQE) is explained by the fact that the interface contact quality is enhanced. The Raman spectrum in Figure 4f shows that the energy band at 1400–1450 cm^−1^ is attributed to the C_α_ = C_β_ stretching vibration of the five-membered ring of PEDOT:PSS shifting to a lower energy and becoming wider [4]. This indicates that the conformation of the PEDOT:PSS chain changes from a curled to a more linear or extended curled structure after the introduction of the DEP coating, which is beneficial to the conductivity of the PEDOT:PSS thin films. On the one hand, it can induce the conformational change of PEDOT:PSS, hence increasing its conductivity. Moreover, the DEP coating produces a compressive stress on the PEDOT:PSS thin films, which effectively improves the physical contact of the Si/PEDOT:PSS interface and improves the passivation effect of PEDOT:PSS on the Si-PNWs substrate. In summary, introducing DEP enables the Si/PEDOT:PSS interface to establish conformal contact, effectively increasing the device FF from 60.98% to 65.11%, and the PCE of the champion device reaches 13.48%.

## 4. Conclusions

We fabricated the Si-PNWs structure, which effectively reduced the light reflectivity while increasing the contact area. Due to surface tension, PEDOT:PSS and Si-PNWs are rather weakly contacted, resulting in the best-performing device only being able to achieve an FF of 60.98%. Our work introduces DEP coatings that do not affect the light absorption of the HSCs while improving the interface contact between PEDOT:PSS and Si-PNWs, leading to an excellent PCE of 13.48% for the DEP-coated devices. DEP coating improved the surface contact quality between the PEDOT:PSS thin films and the textured structure while improving the contact between the PEDOT: PSS layer and the Ag-grids. This provides a novel strategy for the research of Si/PEDOT:PSS HSCs.

## Figures and Tables

**Table 1:** Photoelectric property parameters and $\bar{R}$ **(%)** of silicon solar cells fabricated using different AgNO_3_ concentrations.

Concentration	$\bar{R}$ (%)	J_SC_ (mA/cm^2^)	V_OC_ (V)	FF (%)	PCE (%)
1 mM	8.41	31.35	0.57	59.09	10.69
3 mM	5.79	32.15	0.58	69.96	11.30
5 mM	4.68	33.59	0.59	60.98	12.18
7 mM	5.64	33.19	0.58	58.40	11.23
8 mM 9 mM	6.19 7.28	31.94 31.60	0.57 0.57	56.39 55.04	10.34 9.85

**Table 2:** Photoelectric property parameters and $\bar{R}$ **(%)** of devices with DEP and without DEP coating.

Texture Structure	$\bar{R}$ (%)	J_SC_ (mA/cm^2^)	V_OC_ (V)	FF	PCE (%)
Si-PNWs *w*/*o* DEP	4.68	33.59	0.59	60.98	12.18
Si-PNWs with DEP	3.89	34.06	0.61	65.11	13.48

## Data Availability

We choose to exclude this statement as the study did not report any data.

## Supplementary Materials

The following supporting information can be downloaded online. Figure S1. SEM of different AgNO_3_ concentrations: (a) 8 mM, (b) 9 mM; Figure S2. The XRD of different structure of samples: (a) planar Si, (b) Si pyramid, (c) SiNWs, (d) Si PNWs; Figure S3. reflectance spectra of different silicon textures.
