# Supplementary material for: Improved Interfacial Contact for Pyramidal Texturing of Silicon Heterojunction Solar Cells
Source: Molecules. 2022 Mar 5;27(5):1710. doi: 10.3390/molecules27051710 (PMC8911853; doi:10.3390/molecules27051710)
Supplement: Supplementary file 1 [file molecules-27-01710-s001.zip › molecules-1553945-supplementary.pdf]

Supporting Information

Improved interfacial contact for pyramidal texturing of silicon heterojunction solar cells

Ruijie Dai [1,2,†], Tengzuo Huang [1,2,†], Weijie Zhou [1,2], Jinpeng Yang [1,2], Hua Zhang [1,2], Fayin Yu [1,2], Anran Chen [1,2,*],Feng Wang [1,2], Jin Zhang [1,2,*], Tao Sun [1,2,*] and Longzhou Zhang [2,*]

1 International Joint Research Center for Optoelectronic and Energy Materials, Yunnan University, Kunming, Yunnan 650091, China
2 School of Materials and Energy, Yunnan University, Kunming 650091, China
* Correspondence: authors: 20180071@ynu.edu.cn
† Ruijie Dai and Tengzuo Huang contributed equally to this work, should be considered co-first authors.

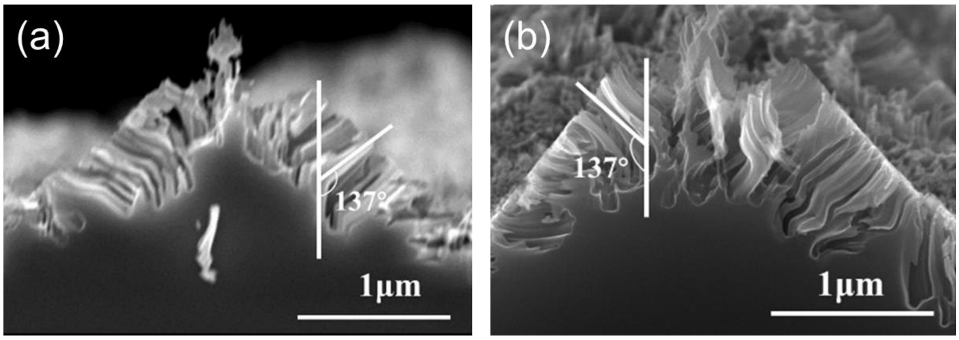

Figure S1. SEM of different $AgNO_3$ concentrations: (a) 8mM, (b) 9mM

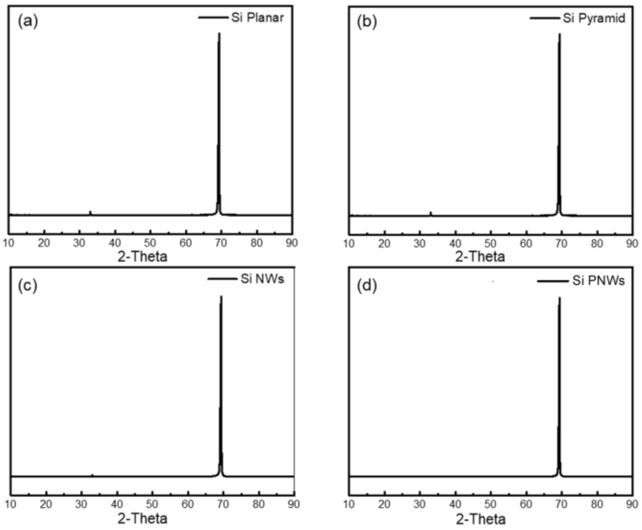

Figure S2. The XRD of different structure of samples: (a) planar Si, (b) Si pyramid, (c) SiNWs, (d) SiNWs/pyramid.

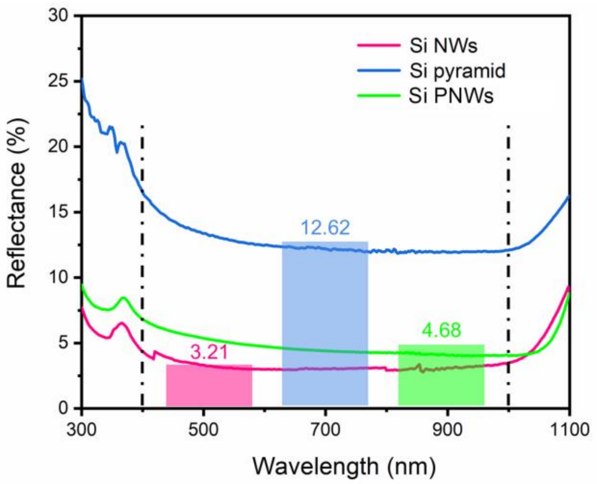

Figure S3. reflectance spectra of different silicon textures.
